# Evaluation of inter-fraction and intra-fraction errors during volumetric modulated arc therapy in nasopharyngeal carcinoma patients

**DOI:** 10.1186/1748-717X-8-78

**Published:** 2013-04-02

**Authors:** Wen-Jing Yin, Ying Sun, Feng Chi, Jian-Lan Fang, Rui Guo, Xiao-Li Yu, Yan-Ping Mao, Zhen-Yu Qi, Ying Guo, Meng-Zhong Liu, Jun Ma

**Affiliations:** 1 State Key Laboratory of Oncology in Southern China, Department of Radiation Oncology, Cancer Center, Sun Yat-sen University, Guangzhou 510060, People’s Republic of China; 2 State Key Laboratory of Oncology in Southern China, Department of Medical Statistics and Epidemiology, Cancer Center, Sun Yat-sen University, Guangzhou 510060, People’s Republic of China

**Keywords:** Cone-beam computed tomography, Setup error, PTV margins, Nasopharyngeal carcinoma, Volumetric modulated arc therapy

## Abstract

**Background:**

This prospective study was conducted to evaluate inter- and intra-fraction errors in nasopharyngeal carcinoma (NPC) patients undergoing volumetric modulated arc therapy (VMAT) using cone-beam computed tomography (CBCT) and to thus obtain planning target volume (PTV) margins to effectively guide treatment in the future.

**Methods:**

Fifteen NPC patients scheduled to undergo VMAT were prospectively enrolled in the study. For each patient, three CBCT scans were obtained; one after daily conventional positioning, one after online correction with 2 mm tolerance and one after 1 week of VMAT delivery. The scans were registered to the planning CT to determine the inter- and intra-fraction errors. Patient positioning errors were analyzed for time trends over the course of radiotherapy. PTV margins were calculated from the systematic (Σ) and random (σ) errors.

**Results:**

The average absolute values of the pre-correction, post-correction and intra-fraction errors (in order) were 1.1, 0.6 and 0.4 mm in the medial–lateral (ML) direction, 1.2, 0.7 and 0.5 mm in the superior–inferior (SI) direction and 1.1, 0.7 and 0.5 mm in the anterior–posterior (AP) direction. The corresponding Σ were 1.0–1.4 mm, 0.4–0.5 mm and 0.2–0.4 mm, while the corresponding σ were 0.7–0.8 mm, 0.6–0.7 mm and 0.5–0.6 mm. With time, gradual increases in both the inter- and intra-fraction three-dimensional displacements were observed (*P* = 0.019 and *P* = 0.044, respectively). The total PTV margins accounting for pre-correction and intra-fraction errors were 3.4–4.1 mm and those accounting for post-correction and intra-fraction errors were 1.7–2.2 mm.

**Conclusions:**

CBCT is an effective modality to evaluate and improve the accuracy of VMAT in NPC patients. Inter- and intra-fraction three-dimensional displacements increased as a function of time during the course of radiotherapy. In our institution, we recommend a PTV margin of 5 mm for NPC patients undergoing VMAT without CBCT and 3 mm for those treated with rigorous daily CBCT scans.

## Background

Nasopharyngeal carcinoma (NPC) is endemic in some regions of the world, especially Southeast Asia. The annual incidence of NPC in Southern China is between 15 and 50 per 100,000 [1]. NPC is unresectable due to the proximity of the tumor to the skull base, and as it has a high radiosensitivity, radiation therapy (RT) remains the mainstay treatment modality for locoregionally confined disease.

Intensity-modulated RT (IMRT) offers superior dose conformity to tumor targets with a relative sparing of critical organs, and recent studies have confirmed that IMRT has a high efficacy of disease control and improved treatment toxicity profile in NPC patients [2,3]. IMRT processes such as posture fixation, computed tomography (CT) simulation and target volume delineation inherently introduce geometrical uncertainties. Errors of even a few millimeters may have a significant impact on dosimetry, due to the steep dose gradients between the tumor and critical organs [4,5]. Hence, to take full advantage of IMRT, it is important to minimize setup error and provide appropriate safety margins around the clinical target volumes (CTVs). To date, few data are available on the quantification of setup error and planning target volume (PTV) margins in NPC patients [5,6].

Intra-fraction motion is one source of positioning error which contributes to the design of PTV margins, and longer treatment times are associated with a greater risk of intra-fraction motion [7]. Only a small number of studies on intra-fraction patient motion monitored by cone-beam CT (CBCT) have been published [8,9]. Furthermore, all of these studies reported errors occurring during IMRT, which requires a long beam delivery time. A recently described novel approach for volumetric modulated arc therapy (VMAT) enables IMRT-like dose distributions with shorter treatment times [10,11]; however, it is unclear how much error occurs during VMAT in NPC patients.

Given the importance of determining the setup error and appropriate PTV expansion for VMAT in NPC patients, we sought to characterize and correct the daily inter-fraction setup errors, as well as the residual errors and intra-fraction errors, using kilovoltage (kV) CBCT. On the basis of the results obtained, we determined appropriate PTV margins with a corresponding formula to account for patient variability during VMAT in NPC patients.

## Methods

### Patient characteristics

We conducted a prospective study on setup measurement error in our center between October 2010 and October 2011. The protocol was approved by the institution’s Protocol Review Board, and all patients provided written informed consent before participation. Eligible patients for this study included individuals with biopsy-proven nonmetastatic NPC, no metal dentures and undergoing definitive-intent VMAT. All patients were staged according to the 7th American Joint Commission on Cancer (AJCC) staging system. Neoadjuvant or adjuvant chemotherapy and concomitant chemotherapy with a platinum-based protocol were recommended for stage III to IVB NPC patients. The characteristics of the patient cohort are shown in Table 1.

**Table 1:** Patient characteristics

**Characteristics**	**No of patients (%)**
**Age**	
Median	44
Range	37-66
**Gender**	
Male	12 (80.0%)
Female	3 (20.0%)
**Histology**	
WHO I	0 (0.0%)
WHO II/III	15 (100.0%)
T stage*	
T1	1 (6.7%)
T2	5 (33.3%)
T3	9 (60.0%)
T4	0 (0.0%)
N stage*	
N0	3 (20.0%)
N1	7 (46.7%)
N2	4 (26.7%)
N3	1 (6.7%)
Clinical stage*	
I	1 (6.7%)
II	2 (13.3%)
III	11 (73.3%)
IV	1 (6.7%)
**Chemotherapy**	
No	3 (20.0%)
Yes	12 (80.0%)

*According to the 7^th^ American Joint Commission on Cancer/Union for International Cancer Control staging system.

### RT simulation and planning

All patients were immobilized using a five-point thermoplastic fixation mask with shoulder fixation (Civco Medical Solutions, Kolona, USA). The target volumes were delineated in accordance with the International Commission on Radiation Units and Measurements reports 50 and 62. The PTVs and planning organ-at-risk volumes were generated by adding a margin of 3 mm to the respective CTVs and corresponding structures such as the spinal cord and brainstem. The prescribed dose was 70 Gy to the PTV of the gross volume of the primary tumor, 64–66 Gy to the PTV of the nodal gross tumor volume, 60 Gy to the PTV of CTV-1 (i.e., high risk regions), 56 Gy to the PTV of CTV-2 (i.e., low-risk regions) and CTV-N (i.e., neck nodal regions) in 33 fractions. Optimization and dose calculation were performed using the Monaco treatment-planning system (version 2.02, Elekta Medical Systems, Crawley, UK) with a Monte Carlo algorithm. All patients were treated with one fraction daily for 5 days per week. Treatment was delivered on a 6-MV linear accelerator equipped with the PreciseBeam VMAT® linac control system (Elekta Medical Systems).

### Image-guided radiotherapy procedure

#### CBCT imaging

**Figure 1:**
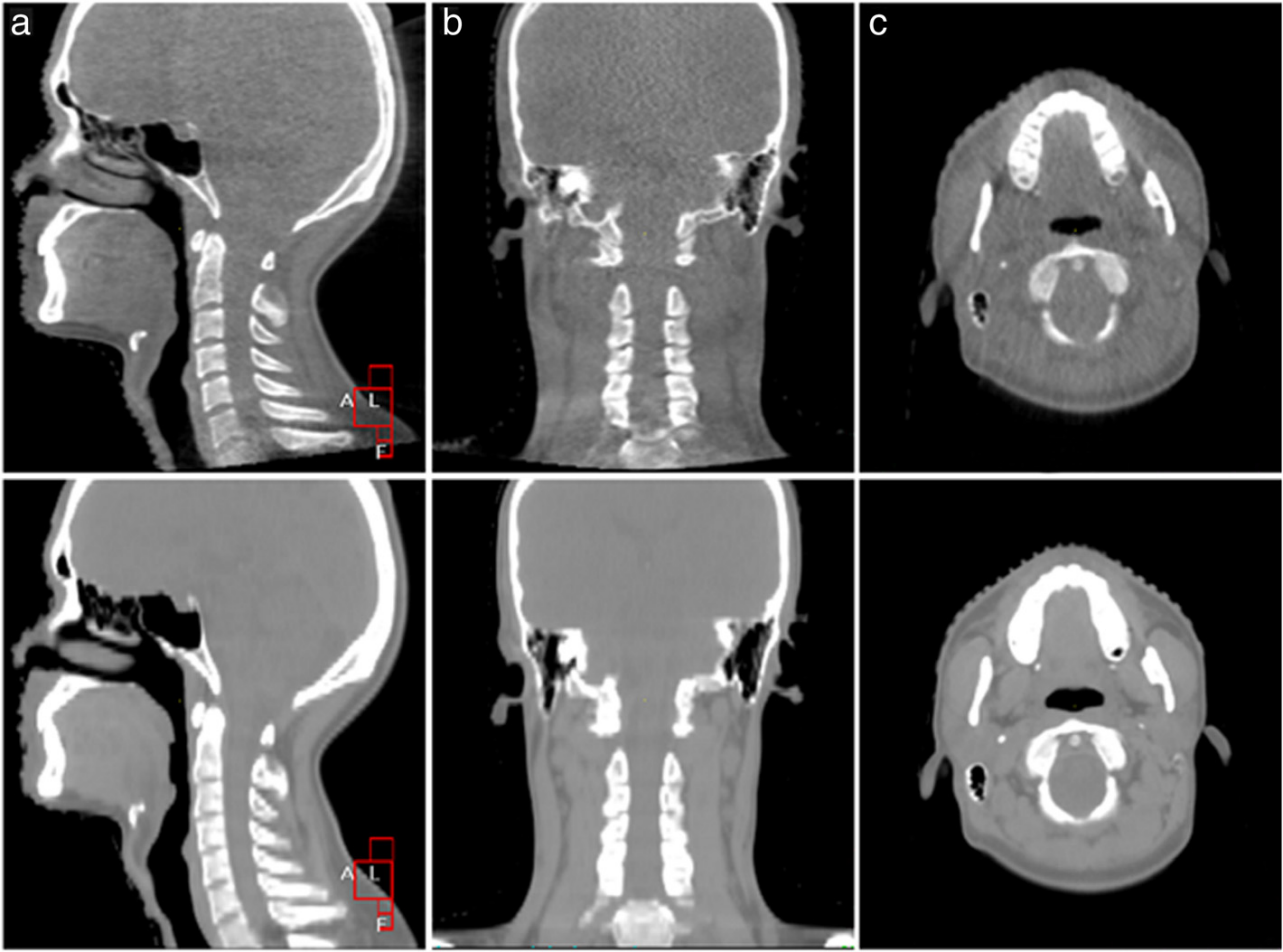
**Comparison of cone-beam computed tomography (CT) images and planning CT images of an NPC patient.** The top and bottom images are cone-beam CT images and planning CT images, respectively. (**a**), (**b**) and (**c**) are images obtained in the sagittal, coronal and transverse planes, respectively.

The kV CBCT images were obtained using the Elekta Medical Systems linear accelerator equipped with kV imaging capabilities (Synergy; Elekta Medical Systems). The acquisition parameters were as follows: kVp, 100 kV; nominal milliamperes per frame, 10 mA; nominal milliseconds, 10 ms; kV collimator, s20; kV filter, f0; approximate frames, 361; and total angle, 200. Figure 1 shows the contrast between CBCT images and planning CT images of the nasopharynx and neck in the sagittal, coronal and transverse sections. It is obvious that the CBCT images have the quality needed to perform an accurate image registration with planning CT images.

#### CBCT guidance protocol

The kV CBCT images were acquired on a daily basis after conventional positioning by aligning the in-room lasers with the marks drawn on the masks. If the translational error was greater than 2 mm in any direction, setup corrections were made by adjusting the patient position through automatically shifting the treatment couch in all three translational dimensions. Then, a second CBCT scan was performed to measure the residual setup error and confirm the accuracy of the automatic correction. If the error was greater than 3° or 5 mm in any direction, the therapists removed and re-fitted the mask on the patient, verifying the correct placement. After VMAT delivery, a final CBCT scan was acquired to assess intra-fraction motion once a week. This imaging schedule was discontinued temporarily if the workload of patient treatment was heavy, or if the CBCT scanner was unavailable on the day of treatment.

### CBCT image registration

All of the acquired images were assessed online by the radiation therapists, by registering the CBCT scan to the planning CT scan by automatic bone matching using Elekta Medical Systems XVI software. No manual adjustments were performed. The alignment box for automatic image registration included the target volume and organs at risk (such as the spinal cord and brain stem) and the surrounding bony structures above the C6 vertebrae.

### Set-up errors protocol

Setup shifts were defined as the deviations between the CBCT and the planning CT in the medial–lateral (ML), superior–inferior (SI) and anterior–posterior (AP) directions, as well as pitch, roll and yaw. The pre-correction CBCT scan acquired after the in-room setup was used to calculate the initial inter-fraction error. The post-correction CBCT scan acquired after any corrections, or the pre-correction CBCT scan for fractions where the initial setup was within ± 2 mm tolerance, was used to calculate the residual inter-fraction error. The difference between the offset of the post-treatment CBCT and the post-correction CBCT, or the difference between the offset of the post-treatment CBCT and the pre-treatment CBCT of less than 2 mm, was used to calculate intra-fraction error.

### Statistical analysis

The mean and standard error of the absolute values of pre-correction, post-correction and intra-fraction errors were calculated. For each patient, the mean (m) and standard deviation (SD) of the daily measurements was calculated. The group mean (M, the mean of all patients’ means), the systematic setup uncertainty (Σ, the standard deviation of M) and the random setup uncertainty (σ, the root mean square of the SD of all patients) were calculated for both inter-fraction and intra-fraction errors [12]. The overall Σ and σ were defined as the square root of the quadratic sum of the inter-fraction and intra-fraction Σ and σ, respectively. To calculate the PTV margins, we followed the geometric margin formula developed by van Herk *et al.*, (2.5 Σ + 0.7 σ), which ensures that the minimum CTV dose is 95% for 90% of patients [13].

We also calculated the three-dimensional (3D) displacement, which was defined as the square root of the quadratic sum of the three components of an error. As inter- and intra-fraction errors may increase as the patient’s weight decreases, we examined the relationship between weight loss and setup errors, and analyzed the time trend of weight loss. We then analyzed the differences in setup errors as a function of time by dividing the RT course into intervals of 11 fractions, which were associated with significant differences in weight. The one-way ANOVA F-test for repeated measurements was used to analyze the changes in the displacement as a function of time for each translational direction, as well as the 3D vector direction. The least significance difference was used to compare the difference in displacements between each time group. The criterion for statistical significance was set at *P* = 0.05, and *P* values were based on two-sided tests. All analyses were performed using SPSS software, version 16.0 (SPSS, IL, Chicago, USA).

## Results

### Number of images and CBCT scan time

A total of 596 CBCT images were acquired from the 15 NPC patients, including 352 pre-correction images (71.1% of fractions), 149 post-correction images (30.1% of fractions) and 95 post-treatment images (19.2% of fractions). The median number of images per patient was 39.

The interval between the initial CBCT and post-correction CBCT was 4.2 min (range, 3.1–8.0 min), while the interval between the post-treatment CBCT and post-correction CBCT was 11.8 min (range, 9.3–16.8 min).

### Inter-fraction error

**Figure 2:**
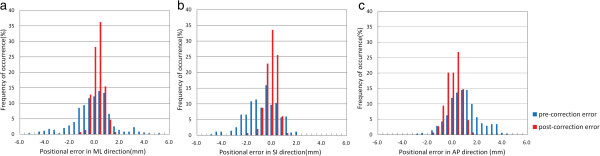
**Distribution of differences between pre- or post-correction cone-beam computed tomography scans and planning CT scans.** (**a**), (**b**) and (**c**) show the distribution of setup errors in the medial–lateral, superior–inferior and anterior–posterior directions, respectively.

The distribution of inter-fractional setup errors in each of the three translational directions were calculated using the 352 pre-correction images and 149 post-correction images (Figure 2). In all directions, the distributions obtained from the post-correction scans were narrower than those obtained from the pre-treatment scans, all lying within the ±2 mm tolerance level for total treatment fractions. The average absolute values of the pre-correction errors were 1.1, 1.2 and 1.1 mm in the ML, SI and AP directions, respectively, while the corresponding values of post-correction errors were 0.6, 0.7 and 0.7 mm. For initial inter-fractional shifts, the number of fractions exceeding ±2 mm in the ML, SI and AP directions were 57 (16.2%), 64 (18.2%) and 53 (15.1%), respectively, and 22 (6.3%), 51 (14.5%) and 19 (5.4%) fractions exceeded 2° for pitch, roll and yaw, respectively. The residual inter-fraction Σ and σ values were significantly smaller than the initial inter-fraction Σ and σ values (Table 2).

**Table 2:** Summary of inter-fraction and intra-fraction translational error and rotational error in each dimension

**Translational error (mm)**									
	**Initial inter-fraction error**			**Residual inter-fraction error**			**Intra-fraction error**		
	**ML**	**SI**	**AP**	**ML**	**SI**	**AP**	**ML**	**SI**	**AP**
M	−0.3	−0.9	0.8	0	−0.4	0.2	0	−0.2	−0.3
SD	1.5	1.3	1.2	0.7	0.8	0.8	0.5	0.7	0.7
Minimum	−5.2	−4.8	−2.9	−2.0	−2.0	−1.9	−1.5	−2.4	−2.8
Maximum	5.0	2.0	4.2	1.8	2.0	2.0	2.1	1.8	0.7
Σ	1.4	1.0	1.0	0.4	0.5	0.4	0.2	0.4	0.4
σ	0.8	0.8	0.7	0.6	0.7	0.7	0.5	0.6	0.6
Rotational error (°)									
	Initial inter-fraction error			Residual inter-fraction error			Intra-fraction error		
	pitch	roll	yaw	pitch	roll	yaw	pitch	roll	yaw
M	−0.7	−0.2	−0.2	−0.7	−0.1	−0.1	−0.1	0.1	0
SD	0.9	1.4	1.0	0.9	1.4	0.9	0.5	0.5	0.4
Minimum	−3.5	−4.4	−4.0	−3.0	−3.0	−3.0	−2.8	−1.2	−1.5
Maximum	2.1	2.9	2.8	1.8	2.9	2.7	1.4	1.5	0.9
Σ	0.7	1.0	0.7	0.7	1.0	0.6	0.3	0.2	0.2
σ	0.6	1.0	0.7	0.6	1.0	0.7	0.5	0.5	0.4

M, mean of all patients’ mean; SD, standard deviation; Σ, systematic setup uncertainty; σ, random setup uncertainty; ML, medial–lateral; SI, superior–inferior; AP, anterior–posterior.

### Intra-fraction error

The mean time interval of intra-treatment motion assessment was 7.3 ± 1.2 min (range, 5.6–9.4 min). No significant correlation was observed between the delivery time and the intra-fraction errors in the ML, SI, AP and 3D directions (*P* = 0.373, 0.523, 0.324 and 0.327, respectively). The mean absolute intra-fraction change was 0.4, 0.5 and 0.5 mm for the ML, SI and AP directions, respectively, while the corresponding values for the pitch, roll and yaw directions were 0.4°, 0.4° and 0.3°, respectively. The number of fractions exceeding ±2 mm in the ML, SI and AP directions were 1 (1.1%), 1 (1.1%) and 4 (4.2%), respectively, and 1 (1.1%), 0 (0.0%) and 0 (0.0%) fractions exceeded 2° for pitch, roll and yaw, respectively. Intra-fraction motion was significantly smaller than the initial inter-fraction error in both the translational and rotational directions (Table 2).

### Displacement as a function of time

**Figure 3:**
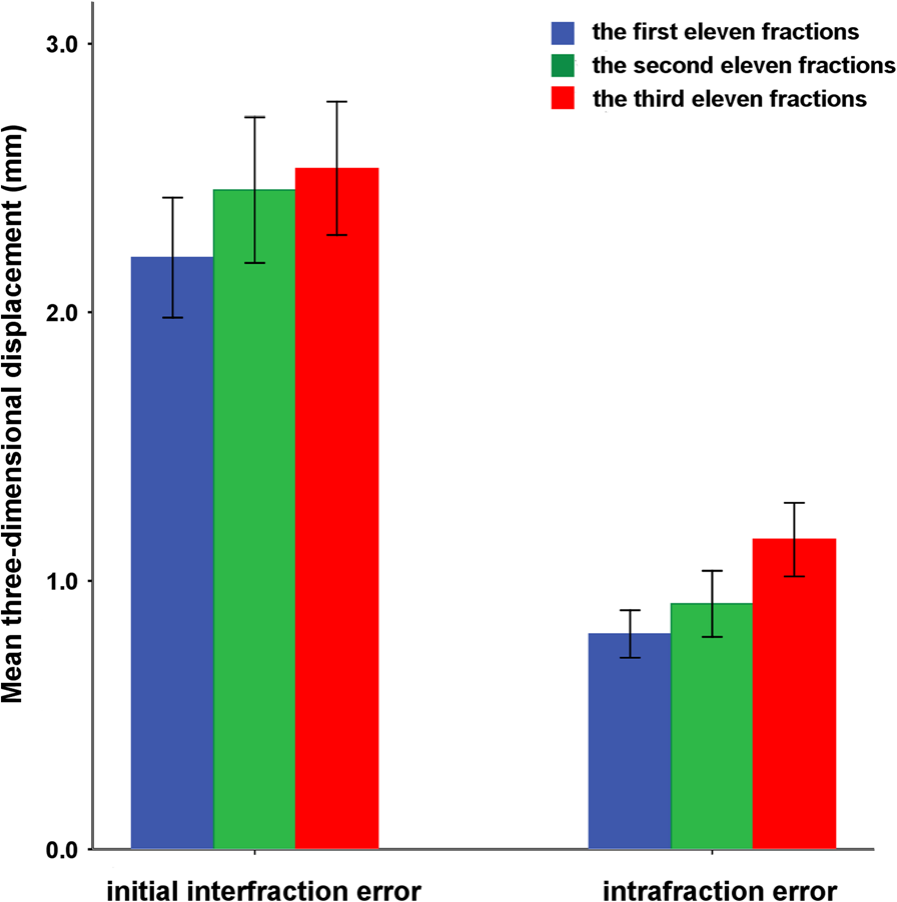
**Inter- and intra-fraction three-dimensional setup errors vary as a function of time.** Error bars represent one standard deviation.

Our results showed that weight loss was significantly correlated with inter-fraction errors in the ML and 3D directions and with the intra-fraction error in the 3D directions (*P* < 0.05). The weight decreased significantly when the total treatment time was divided into three equal sections of 11 fractions each (*P* < 0.05). We, therefore, calculated the mean displacement for each patient after every 11 treatment fractions. The initial inter-fraction errors in the ML and AP directions and the intra-fraction errors in the AP and SI directions gradually increased, but no statistical difference was observed during the treatment period (*P* = 0.315, 0.065, 0.177 and 0.220, respectively). A gradual increase in the initial inter-fraction and intra-fraction 3D displacements was observed as a function of time (*P* = 0.019 and *P* = 0.044, respectively; Figure 3). Further comparisons of displacements in the three time phases indicated that both the initial inter-fraction and intra-fraction 3D displacement values were significantly higher in the last 11 fractions than in the first 11 fractions (2.2 mm vs. 2.5 mm, *P* = 0.003; 0.8 mm vs. 1.2 mm, *P* = 0.025).

### PTV margins

For all patients, a comparison of the PTV margins obtained from pre-correction, post-correction and post-treatment CBCT images showed that PTV margins can be significantly reduced with the online correction, and margins accounting for intra-fraction errors were smaller than those account for the initial inter-fraction errors (Table 3). The total margins accounting for initial inter-fractional and intra-fractional setup errors were 4.1, 3.4 and 3.5 mm in the ML, SI and AP directions, respectively, in contrast to the corresponding required target margins of only 1.7, 2.2 and 2.2 mm in each of the three directions, if the residual and intra-fraction errors were considered after online correction.

**Table 3:** Planning target volume margins in each translational direction

	**ML (mm)**	**SI (mm)**	**AP (mm)**
Initial inter-fraction	4.0	3.1	3.1
Residual inter-fraction	1.5	1.7	1.6
Intra-fraction	0.9	1.4	1.5
Total without CBCT correction	4.1	3.4	3.5
Total with CBCT correction	1.7	2.2	2.2

ML, medial–lateral; SI, superior–inferior; AP, anterior–posterior; CBCT, cone beam computed tomography.

## Discussion

CBCT provides a promising method to quantify inter-fraction and intra-fraction errors, and allows a significant reduction in inter-fraction errors. To our knowledge, this is the first report on intra-fraction errors during VMAT in NPC patients, and it provides a guide for designing PTV margins in NPC patients during VMAT.

### Inter-fraction error

When applying new treatment techniques to malignancies at other sites, it is necessary to determine setup errors to acquire appropriate PTV margins. The inter-fraction error observed in this study is similar to the inter-fraction error reported by other researches, including the study of Wang *et al.* in which CBCT analysis indicated that the systematic deviations and random errors during set-up in NPC patients were 1.1–1.3 mm [5]. Similarly, Velec *et al.* analyzed daily CBCT images acquired during the treatment of 20 head and neck cancer patients, and obtained systematic deviations ranging from 0.8 mm to 1.1 mm and random translational deviations of less than 2 mm [9]. However, a review carried out by Hurkmans *et al.* focusing on set-up verification in head and neck cancer patients using portal imaging, concluded that the systematic and random deviations varied by 1.6–4.6 mm and 1.1–2.5 mm, respectively [14], which are higher than the values in this study. These differences may be due to the fact that we automatically matched a smaller region of interest confined to the nasopharynx and upper neck, as there is evidence that positional variation is greater in the lower neck than in the upper neck [15]. In addition, our study used rigorous immobilization devices, such as the thermoplastic mask covering the head, neck and shoulders, and such devices have previously been used in IMRT delivery for head and neck cancer to improve the reproducibility and stability of patient position [16].

In our study, CBCT effectively improved the accuracy of VMAT in NPC patients because the inter-fraction systematic error could be decreased from 1.0–1.4 mm to 0.4–0.5 mm, which was similar to the results reported by Wang *et al.* and Dionisi *et al.*[5,17]. While the uncertainties in delivering RT can be reduced with CBCT systems, it is important to understand the uncertainties arising from this process. One of the important factors is the uncertainties in image registration. It is current practice in head and neck cancer to use rigid registrations of bony anatomies with the planning CT, to obtain setup errors. The accuracy of these registrations can be affected by the image dose, image resolution, region of interest used for registration *etc.*[18-20]. Many research studies have reported bone alignment with an accuracy of nearly 1 mm for the translational displacement of a head phantom [21,22]. However, head and neck cancer patients experience significant deformation, shrinkage and rotation, all of which could also affect the efficacy of a bone match. Recent studies have shown that due to the considerable and frequent shape and posture changes in patients with head and neck cancer, not all structures within a single large region of interest can be simultaneously aligned using bone registration [23,24]. Therefore, misregistration can occur and the misalignment can persist due to shape or posture changes. It is encouraging that new registration and correction methods to reduce such misalignment and deformation have been proposed [25,26]. In future, software updates will be aimed at automating these methods to allow better quantification of the setup error.

Furthermore, due to the limited availability of conventional treatment tables, not all users of the bone registration algorithm can adjust the rotational setup of the patient to minimize setup error [9,27]. There is a possibility of significant misalignment when only the translational part of a six-dimensional (6D) registration is applied to the patient’s position in a completely general 6D registration framework. Fortunately, strategies for avoiding this misalignment have been proposed by Murphy [28]; these consist of putting the landmark/treatment isocenter at or very near the origin of the rotation axes or calculating only a 3D registration using landmarks near the treatment site. Therefore, it is important to realize the limits of bone registration and deal carefully with the practical application of bone registration tools and patient setup practices during imaging.

### Intra-fraction error

Generally, intra-fraction error was mainly influenced by the immobilization device and delivery time. Theoretically, the magnitude and probability of patient intra-fraction movement will most likely increase when fraction times are extended. Hoogeman *et al.* concluded that intra-fraction systematic geometric error increases with time [7]. However, our study showed that there was no significant correlation between the delivery time and intra-fraction error. One reason for this result was possibly the narrow range of delivery time (5.6–9.4 min), which did not allow statistically significant results to be obtained. Another reason was that limited data were available for analysis, as our study included only 15 patients and six CBCT scans for each patient. In addition, in our study, the intra-fraction systematic error ranged from 0.2 mm to 0.4 mm during the 5–9 min VMAT time. Velec *et al.* reported an intra-fraction systematic error of 0.3–0.7 mm in patients fixed with a thermoplastic mask covering the head, neck and shoulders during an approximately 15-min IMRT time [9]. A comparison study of IMRT and VMAT in the same institution is required to determine whether VMAT is associated with reduced intra-fraction motion.

Patient intra-treatment movement can be assessed by several methods along a continuum of imaging frequencies. The most commonly used method of assessing intra-treatment motion is pre- and post-treatment imaging [8,9], which we used in this study. The other approaches are intermittent imaging, acquired as frequently as every 0.5–2 min, or continuous, real-time tracking of the tumor target during radiation delivery with technologies such as CyberKnife or electromagnetic localization [7,29]. However, as motion may be sustained during the entire course of radiation delivery, differences in the measurement and acquisition schedule could potentially lead to discrepancies between the measured motion and actual motion at radiation delivery. Further studies using continuous imaging should be performed to evaluate the intra-fraction motion during radiation delivery in NPC patients undergoing VMAT.

### Gradual increase in displacement as a function of treatment time

Treatment accuracy during fractionated radiotherapy may decrease with time due to tumor regression or weight loss [30]. Den *et al.* reported that PTV margins in the last 3 weeks were significantly larger than those in the first 3 weeks [27]. We obtained a similar result, as patient inter-fraction and intra-fraction 3D displacements increased gradually as a function of time. Adaptive re-planning strategy is an effective method to account for significant dosimetric variation during radiotherapy, which was mainly caused by setup errors and anatomical changes. Currently, the optimal timing of re-planning remains to be determined.

### Appropriate PTV margins

Reasonable designs of PTV margins are the key point for local control and normal tissue protection. Narrow margins tend to be associated with local recurrence, while wide margins result in excessive treatment. Decreasing the PTV margins can theoretically improve the therapeutic gains; such a benefit was illustrated by van Asselen *et al.*[31], who observed that narrow PTV margins improved parotid sparing and decreased the probability of normal-tissue complications such as xerostomia. However, the application of narrow margins must be based on the premise of excellent quality-control measures such as daily CBCT online correction. In general, narrow margins are not widely used in clinical practice, and are reserved for special cases such as locally advanced tumors that invade tissues adjacent to the brain stem or spinal cord. In this study, we have discussed the PTV margins accounting for setup errors, and these margins were 1.7–2.2 mm and 3.4–4.1 mm with and without correction, respectively. However, it should be noted that PTV margins are not the only component of the setup margin (inter- and intra-fraction motion), and other process-related components such as image registration, treatment planning uncertainties and transfer errors from the planning CT to the simulator should be accounted for. In our institution, therefore, we recommend a PTV margin of 5 mm for NPC patients undergoing VMAT without CBCT and 3 mm for those treated with rigorous daily CBCT scans. Our results provide a theoretical basis for the appropriate design of PTV margins for VMAT in NPC patients.

## Conclusions

CBCT is an effective modality to evaluate and improve the accuracy of VMAT in NPC patients. Inter- and intra-fraction 3D displacements increased as a function of time during the course of radiotherapy. In our institution, we recommend a PTV margin of 5 mm for NPC patients undergoing VMAT without CBCT and 3 mm for those treated with rigorous daily CBCT scans.

## Competing interests

The authors indicate no actual or potential conflicts of interest exist.

## Authors’ contributions

The authors contributions are the following: W-JY and YS contributed to literature research, study design, data collection, data analysis, interpretation of findings and writing of the manuscript. FC and J-LF contributed to image registration and data collection. RG and X-LY contributed to patient recruitment, data collection and data analysis. Z-YQ and M-ZL contributed to study design and carried out the quality assurance. YG (PhD, professor) contributed to data analyses. JM contributed to data collection, study design, critical review of data analyses, interpretation of findings and critical edit of the manuscript. All authors read and approved the final manuscript.
